# Massive foreign body reaction and osteolysis following primary anterior cruciate ligament reconstruction with the ligament augmentation and reconstruction system (LARS): a case report with histopathological and physicochemical analysis

**DOI:** 10.1186/s12891-022-05984-5

**Published:** 2022-12-30

**Authors:** Luca Ambrosio, Gianluca Vadalà, Rachele Castaldo, Gennaro Gentile, Lorenzo Nibid, Carla Rabitti, Luigi Ambrosio, Edoardo Franceschetti, Kristian Samuelsson, Eric Hamrin Senorski, Rocco Papalia, Vincenzo Denaro

**Affiliations:** 1 Operative Research Unit of Orthopaedic and Trauma Surgery, Fondazione Policlinico Universitario Campus Bio-Medico, Via Alvaro del Portillo 200, 00128 Rome, Italy; 2 Research Unit of Orthopaedic and Trauma Surgery, Department of Medicine and Surgery, Università Campus Bio-Medico di Roma, Via Alvaro del Portillo 200, 00128 Rome, Italy; 3 Institute of Polymers, Composites and Biomaterials, National Research Council, Naples, Italy; 4 Department of Human Pathology, Fondazione Policlinico Universitario Campus Bio-Medico, Rome, Italy; 5 Department of Orthopaedics, Institute of Clinical Sciences, Sahlgrenska Academy University of Gothenburg, Gothenburg, Sweden; 6 Sahlgrenska Sports Medicine Center, Gothenburg, Sweden; 7 Department of Orthopaedics, Sahlgrenska University Hospital, Mölndal, Sweden; 8 Unit of Physiotherapy, Department of Health and Rehabilitation, Institute of Neuroscience and Physiology, Sahlgrenska Academy, University of Gothenburg, Gothenburg, Sweden

**Keywords:** LARS, ACL, Ligament, Reconstruction, Case report, Complications, Synthetic, Graft

## Abstract

**Background:**

Autologous hamstrings and patellar tendon have historically been considered the gold standard grafts for anterior cruciate ligament reconstruction (ACLR). In the last decades, the utilization of synthetic grafts has re-emerged due to advantageous lack of donor site morbidity and more rapid return to sport. The Ligament Augmentation and Reconstruction System (LARS) has demonstrated to be a valid and safe option for ACLR in the short term. However, recent studies have pointed out the notable frequency of associated complications, including synovitis, mechanical failure, and even chondrolysis requiring joint replacement.

**Case presentation:**

We report the case of a 23-year-old male who developed a serious foreign body reaction with wide osteolysis of both femoral and tibial tunnels following ACLR with LARS. During first-stage arthroscopy, we performed a debridement of the pseudocystic mass incorporating the anterior cruciate ligament (ACL) and extending towards the tunnels, which were filled with autologous anterior iliac crest bone graft chips. Histological analysis revealed the presence of chronic inflammation, fibrosis, and foreign body giant cells with synthetic fiber inclusions. Furthermore, physicochemical analysis showed signs of fiber depolymerization, increased crystallinity and formation of lipid peroxidation-derived aldehydes, which indicate mechanical aging and instability of the graft. After 8 months, revision surgery was performed and ACL revision surgery with autologous hamstrings was successfully carried out.

**Conclusions:**

The use of the LARS grafts for ACLR should be cautiously contemplated considering the high risk of complications and early failure.

**Supplementary Information:**

The online version contains supplementary material available at 10.1186/s12891-022-05984-5.

## Background

Graft choice for anterior cruciate ligament reconstruction (ACLR) remains a topic of high debate. Indeed, several long-term studies and systematic reviews have reported excellent clinical outcomes and survivorship with the use of hamstring and patellar tendon grafts [1]; furthermore, the quadriceps tendon has recently gained attention as a viable and efficacious alternative [2]. However, these solutions are affected by well-known complications such as the risk of donor site morbidity (including bleeding, persistent pain, infection, etc.) and prolonged rehabilitation. Nonetheless, autografts still remain the ‘gold standard’ in primary ACLR [3]. Therefore, a great interest towards the development of synthetic anterior cruciate ligament (ACL) grafts that may overcome such drawbacks has been raised in the last decades. Notwithstanding, earlier attempts in 1970s were burdened by serious issues such as poor clinical results, a high rate of mechanical failures as well as post-operative synovitis and premature osteoarthritis (OA) [4]. The Ligament Augmentation and Reconstruction System (LARS, Surgical Implants and Devices, Arc-sur-Tille, France) is a polyethylene terephthalate (PET) synthetic ACL graft firstly introduced in early 1990s and promoted as being able to consent a rapid return to sport while eliminating the morbidity of autologous tissue harvest and reducing the risk of synovitis compared to previous grafts [5, 6]. Although the first studies investigating short- to medium-term outcomes for the LARS ligament showed comparable results to autologous ACL grafts and low failure rates [4, 7, 8], more recent reports displayed a considerably high degree of failure at the long term, reaching 31–50% at 6–10 years [9–11]. Complications related to LARS have repeatedly been reported in the literature and include mechanical failure [9], acute and chronic synovitis [12], foreign body reaction [13], bony tunnel enlargement [14], and even early OA requiring total knee replacement (TKR) [15].

We present a report of mechanical failure and disabling knee pain following primary ACLR using LARS artificial ligament that required revision surgery due to a severe foreign body reaction with the development of synovitis and a pseudo-myxoid mass within the intercondylar notch and bony tunnel osteolysis.

## Case presentation

A moderately active 23-year-old warehouseman (body mass index: 22.2 kg/m^2^) with no relevant medical, family, genetic, and psychosocial history was referred to our clinic with complaints of pain and functional instability of the right knee. Five years before, the patient ruptured his ACL during an occasional soccer match and underwent right primary ACLR with LARS at another institution. Immediately after surgery, he started isometric quadriceps strengthening, hamstring stretching and strengthening, hip adduction and abduction and progressive range of motion (ROM) restoration until reaching 0–100° (extension/passive flexion) at day 10. After 2 weeks, stationary bike, closed chain exercises and gradual restoration of active full ROM were initiated. At 1 month postoperatively, open chain exercises and progressive return to previous activities were indicated. After biweekly visits during the first month postoperatively, the patient was followed up every 3 months for 1 year.

**Fig. 1:**
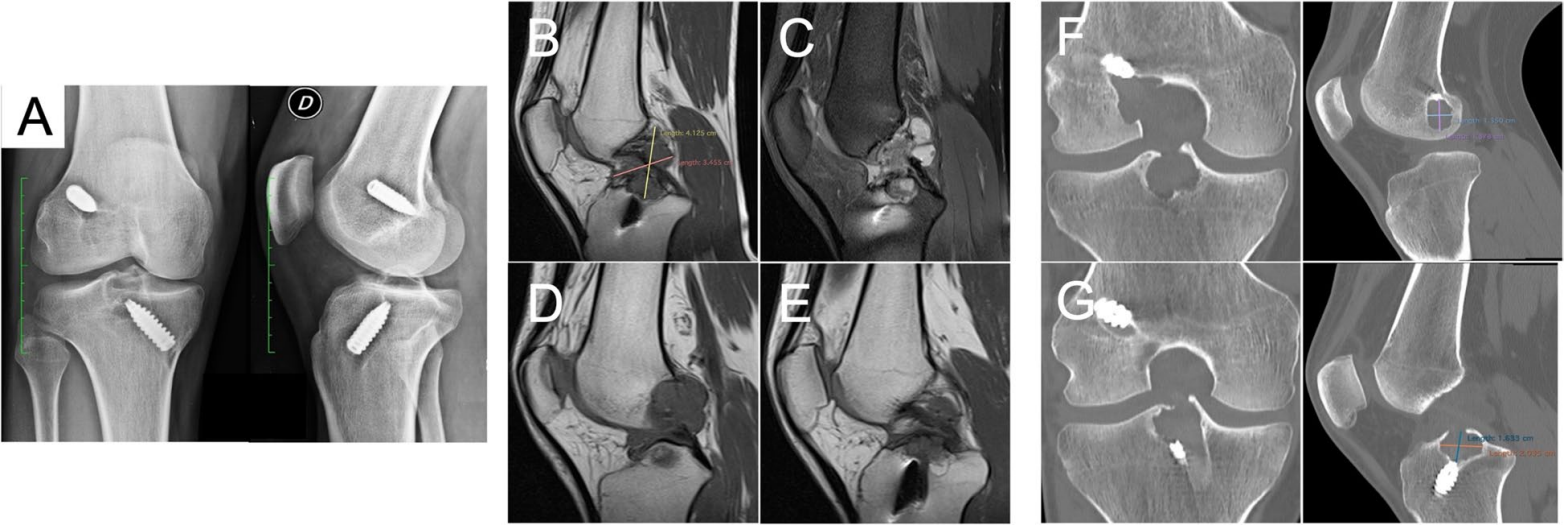
**A**, preoperative X-ray exam showing tunnel enlargement and osteolytic resorption of the intercondylar eminence. B-E, MRI representative images showing the size of the pseudo-myxoid mass occupying the intercondylar notch (**B**). STIR images demonstrated the presence of concamerated fluid cysts within the mass with no clear signs of the ACL graft (**C**). The lesion expanded both towards the femoral (**D**) and tibial tunnels (**E**). F-G, CT representative images showing the morphology and maximum diameter of the femoral (**F**) and tibial (**G**) osteolytic cavities surrounding the tunnels

**Fig. 2:**
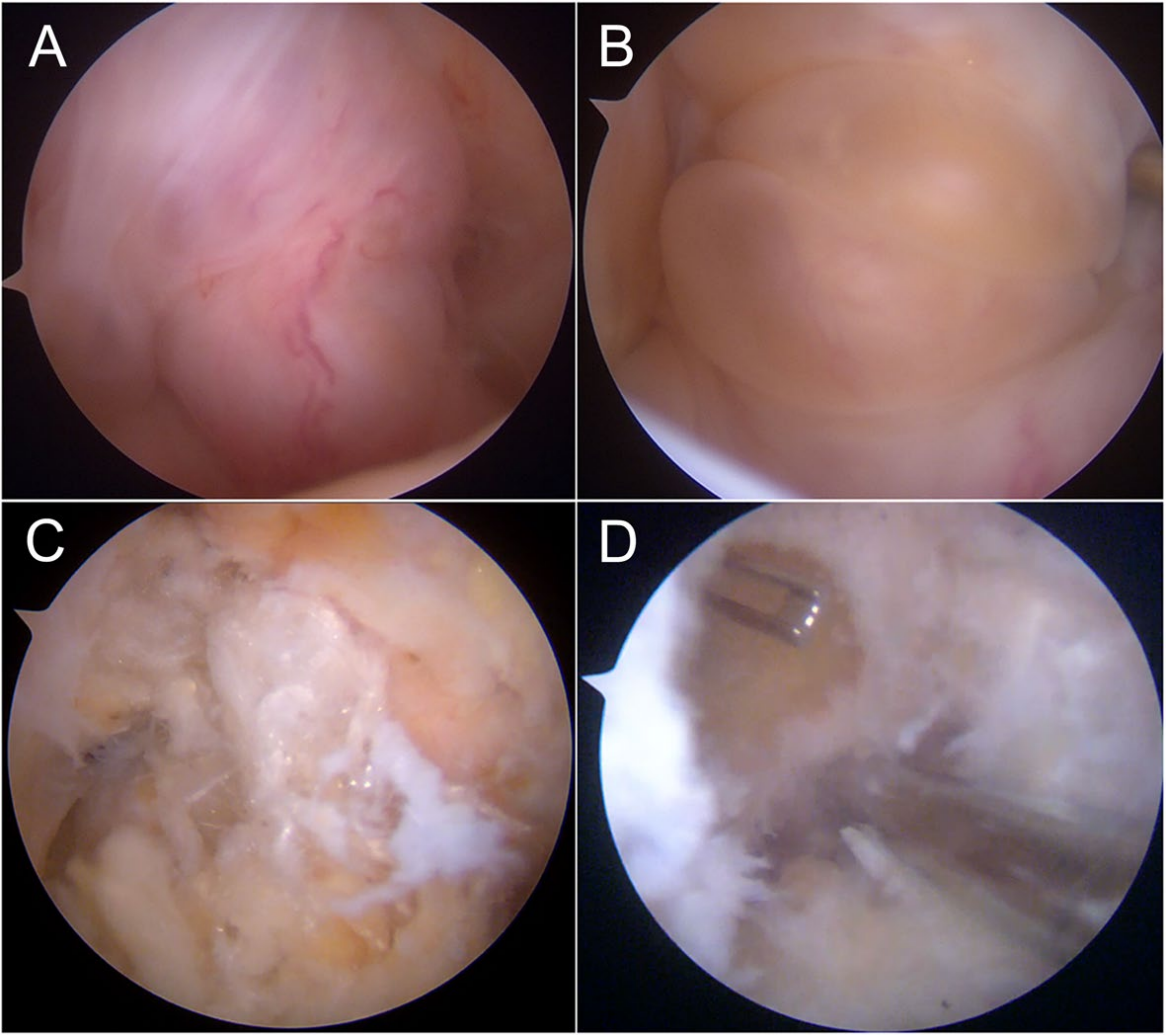
Arthroscopic intraoperative representative images showing the presence of a consistent, highly vascularized mass occupying the intercondylar notch and embracing the LARS graft (**A**). By manipulating the lesion with a probe, two posterior cysts appeared (**B**). The central mass was carefully debrided with a shaver, revealing the presence of damaged and elongated synthetic graft fibers (**C**). The tissue was removed from both femoral and tibial tunnels until reaching previously implanted screws (**D**, femoral cavity)

Despite having undergone surgery, the patient still lamented persistent pain even during daily walks and never returned to sport. No history of new trauma was reported following index surgery. On examination, joint effusion was present, and ROM was limited at 110° of active flexion, which was painful. Furthermore, Lachman test, pivot shift and anterior drawer tests were positive. No signs of meniscal injury were retrieved. X-rays demonstrated an enlargement of both femoral and tibial tunnels with increased radiolucency of the intercondylar eminence (Fig. 1A). Magnetic resonance imaging (MRI) showed the presence of a mass embracing the synthetic graft, measuring approximately 4 × 3.5 cm of maximum diameter, and showing hypointensity at T1-weighted images (Fig. 1B) and a pseudo-myxoid, fluid-filled, septated and hyperintense appearance at short tau inversion recovery sequences (STIR, Fig. 1C). Moreover, the mass displayed to extend towards the femoral and tibial tunnel, which showed clear signs of enlargement and osteolytic resorption (Fig. 1D-E). Computed tomography (CT) was ordered to better define the morphology of the osteolytic cavities, with the femoral and tibial defects measuring approximately 1.9 × 1.4 cm (Fig. 1F) and 2 × 1.6 cm (Fig. 1G) of maximum diameter, respectively. All DICOM files were visualized and elaborated using the Horos™ viewer.

According to the study by Mitchell et al. [16], considering suboptimal bone stock, loss of tunnel containment, a tunnel aperture greater than 14 mm and inability to anatomically place or secure an autologous ACL graft during the initial revision procedure, we decided to perform a 2-stage revision ACLR.

### First-stage surgery

**Fig. 3:**
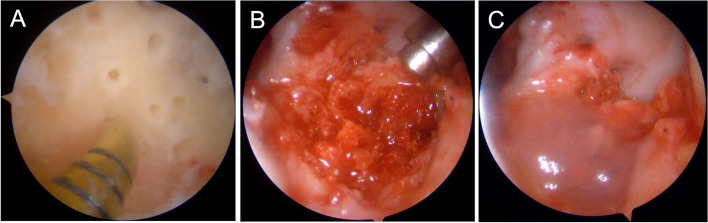
Microfractures of the bone cavities were performed until seeing bleeding bone (**A**, femoral cavity). The cavities were then filled with autologous bone graft harvested from the ASIS (**B**, tibial cavity) and sealed with fibrin glue (**C**, tibial cavity)

Preoperative routine blood tests were performed and did not show evident abnormalities (Supplementary Table 1). Surgery was performed under locoregional anesthesia with the patient in supine position and the right lower limb in a leg holder. Conventional anterolateral and anteromedial arthroscopic portals were established. Within the intercondylar notch a reddish, enlarged and significantly vascularized mass incorporated the ACL graft (Fig. 2A). When the mass was manipulated with a probe, two big cysts were visualized (Fig. 2B). The mass was then gradually removed with a shaver, with elongated and interrupted fibers of the LARS graft appearing at the center of the lesion (Fig. 2C). The synthetic graft and surrounding tissue were carefully debrided from the intercondylar notch and enlarged cavities. The LARS graft was completely removed, while shavers and radiofrequency were used to remove all the remaining tissue until visualizing the tip of previously implanted metallic screws at the extremities of both femoral and tibial tunnels (Fig. 2D). Multiple microfractures of the subchondral bone were performed inside the cavities until bleeding bone and marrow fat droplets were clearly visualized (Fig. 3A). Subsequently, through a 3-cm incision centered on the anterior superior iliac spine (ASIS), a tricortical bone graft was harvested, morcellized in minute bone chips, and mixed with the patient’s own blood. An absorbable gelatin sponge wrapped was used to fill up the iliac crest bone defect and bone wax was utilized to seal the gap and reduce bleeding. A drainage was then positioned. The bone graft was packed into the femoral and tibial bone cavities (Fig. 3B) and sealed with fibrin glue (Fig. 3C). The arthroscopic procedure is available as a video [see Additional file 1]. At the end of the procedure, two samples of synovium and one sample of the LARS graft were sent for histopathological analysis, while the remainder of the graft was utilized for microscopic, Fourier-Transform Infrared (FTIR) and calorimetric analysis. The patient was discharged on the next day with the prescription of walking with two crutches and weight-bearing as tolerated.

### Histological evaluation

**Fig. 4:**
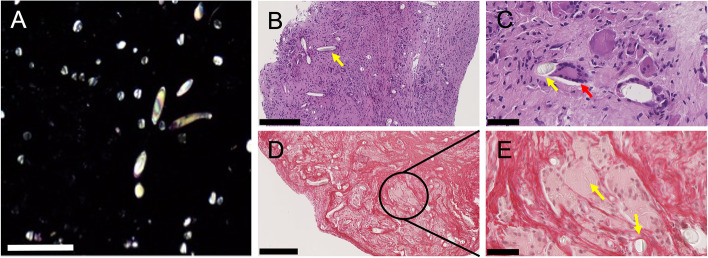
**A**, polarized light microscopy (10X) showing bi-refractive PET on a black background of non-refractive tissue. Scale bar = 250 μm. B-C, H&E staining. Bundles of PET surrounded by reactive fibrosis and chronic inflammation with giant multinucleated cells (**B**, 10x). Scale bar = 250 μm. PET bundle approached by a group of giant multinucleated cells (**C**, 40x). Scale bar = 50 μm. D-E, Sirius Red staining. Positive staining in fibrous tissue (internal control) and negative stain in bi-refractive extra- and intra-cellular bundles and fibers (yellow arrows). **D**, 10x magnification, scale bar = 250 μm. E, 40x magnification, scale bar = 50 μm. Yellow arrows indicate PET fibers. The red arrow indicates giant multinucleated cells

Surgical samples were collected at the Pathology Laboratory of Fondazione Policlinico Universitario Campus Bio-Medico (Rome, Italy). Samples were formalin-fixed and paraffin-embedded; then, 5 μm-sections and hematoxylin-eosin (HE) staining were performed. Each slide was evaluated both at optical and polarized light microscopy applying a polarizer (Nikon C-SP Simple Polarizer, Tokyo, Japan) to the Nikon ECLIPSE Ci-L microscope. All slides were digitalized (NanoZoomer 2.0 RT, Hamamatsu, Japan) at 40x magnification. Optical microscopy revealed wide fibrosis and inflammation with Langhans and foreign-body giant cells. Polarized light microscopy showed bi-refractive foreign material, a typical feature of PET (Fig. 4A). Several patterns of PET were distinguishable in our samples: (i) coarse bundles within the fibrosis, occasionally (ii) surrounded by giant cells or absorbed as (iii) fine fibers into macrophages (Fig. 4B-C). Since collagen fibers also can show bi-refraction under polarized light, we performed a collagen staining (Sirius Red) to confirm the synthetic origin of fibers absorbed by macrophages [17]; negative staining in extra- and intra-cellular bi-refractive bundles and fibers supported the PET nature of the material (Fig. 4D-E).

### Morphological evaluation

**Fig. 5:**
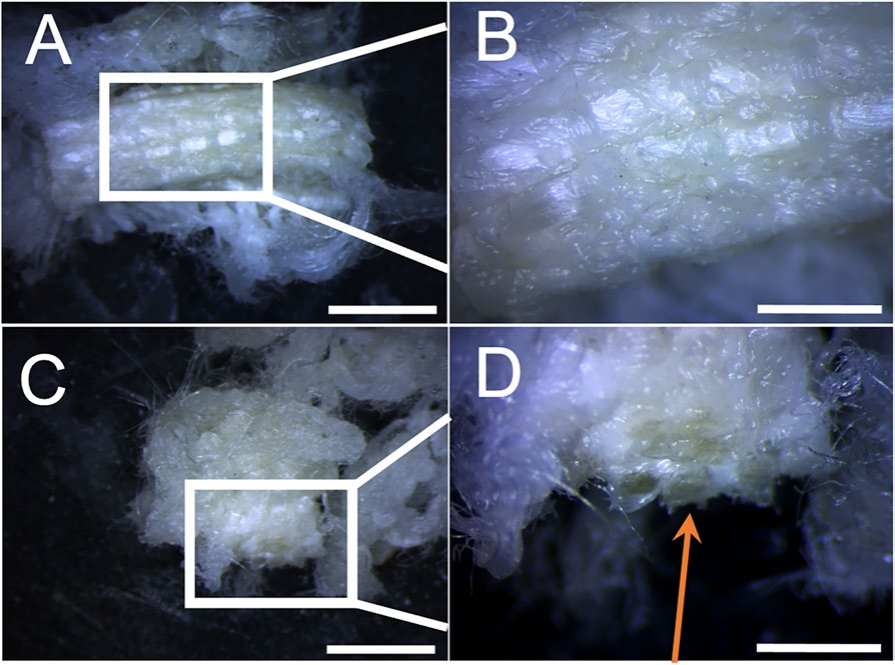
Stereomicroscopic images of the central, tubular shaped part of the explanted ligament (**A**-**B**). The external, rolled up-shaped part of the explanted ligament (**C**), showed the presence of infiltrating biological material, as indicated by the orange arrow (**D**). A, C: scale bar = 5 mm; B, D: scale bar = 2 mm

**Fig. 6:**
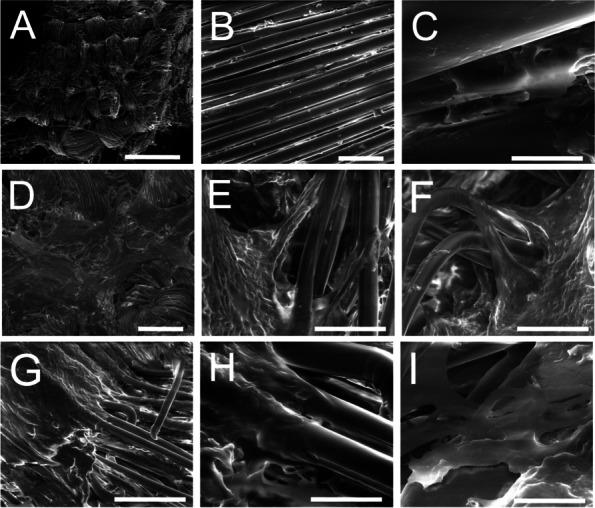
SEM images of the central part of the explanted ligament (upper row, **A**: scale bar = 2 mm; **B**: scale bar = 100 μm; **C**: scale bar = 20 μm) and the external part of the explanted ligament (middle and lower rows, **D**: scale bar = 500 μm; **E** and **F**: scale bar = 100 μm; **G**: scale bar = 200 μm; **H**: scale bar = 50 μm; **I** = scale bar: 20 μm) confirming the large amount of fibrous tissue ingrowth, infiltrating the woven structure of the artificial ligament

The artificial ligament was observed with a Lynx EVO stereomicroscope (Vision Engineering Ltd., Milan, Italy). Sample morphology was analyzed using a FEI Quanta 200 FEG scanning electron microscope (SEM) (FEI, Eindhoven, Netherlands) in low vacuum mode at P_H2O_ = 0.60 Torr, with 10–20 kV acceleration using a large field detector. At stereomicroscopic observation (Fig. 5), two parts of the sample were identified: 1) a central part with a tubular shape (Fig. 5A-B); 2) an external part, with a rolled-up shape (Fig. 5C-D) and presence of infiltrating biological material, as indicated by the orange arrow in Fig. 5D. At SEM observation (Fig. 6), the two parts of the explanted ligament showed different morphology. The central region (Fig. 6A-C) presented an almost unaltered woven structure, with the presence of perpendicular yarns constituted by single fibers with 20–25 μm diameter. As shown, the fiber surface in this region was almost clean. On the contrary, in the external region (Fig. 6D-I), large amounts of residual biological tissue covered the surface of the filaments, well adhering to them, and confirming fibrous tissue ingrowth infiltrating the woven structure of the artificial ligament.

### FTIR analysis

**Fig. 7:**
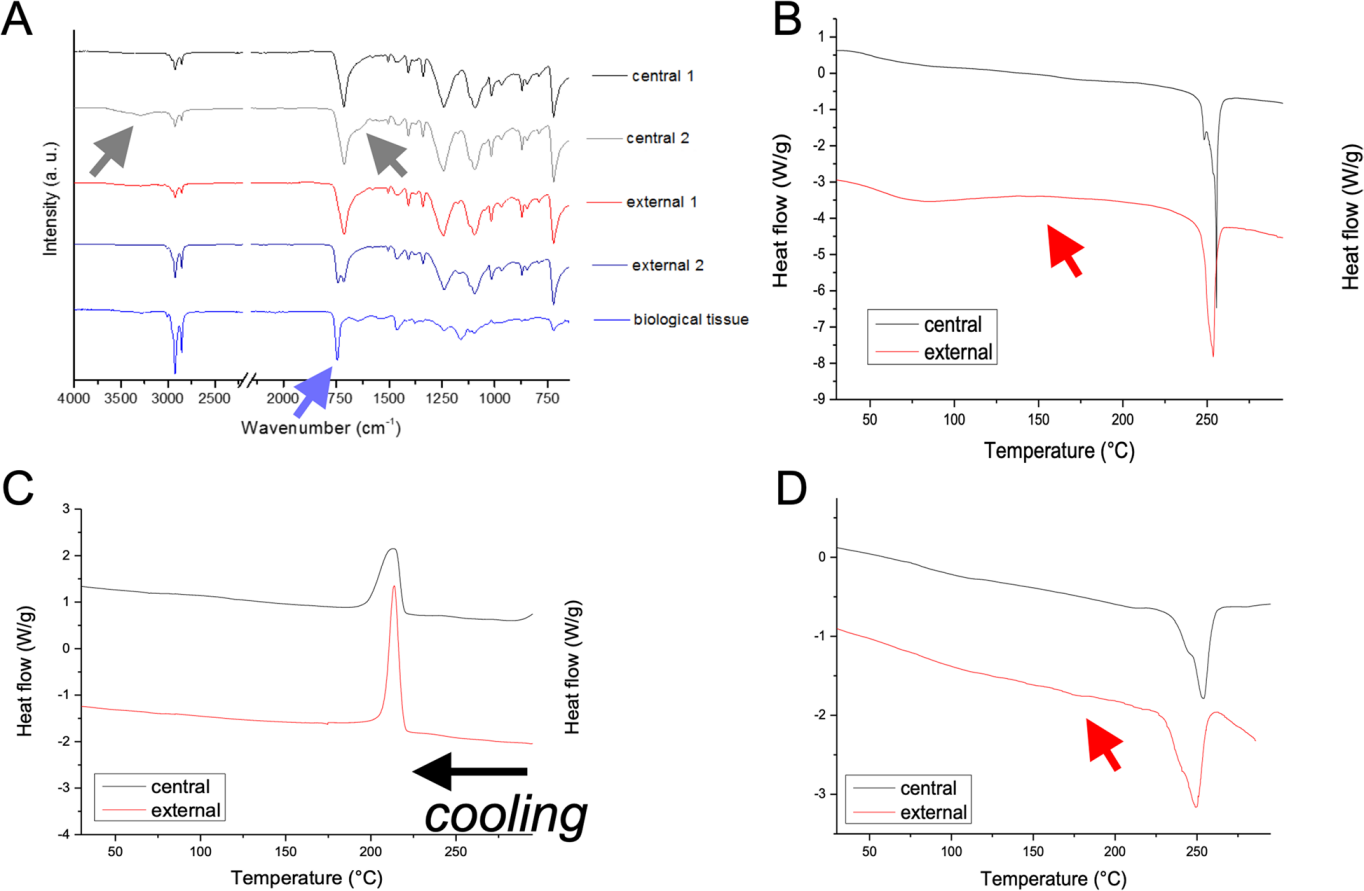
**A**, ATR-FTIR spectra collected in different regions of the central and external parts of the explanted artificial ligament. The bottom spectrum was collected on tissue residues (yellow region evidenced in Fig. 5D). **B**-**D**, DCS traces of the central and external part of the explanted ligament; A) I heating run; B) cooling run; C) II heating run

Attenuated Total Reflectance FTIR (t-FTIR) analysis was performed on ligament samples using a Spectrum One FTIR spectrometer equipped with a Universal ATR accessory (Perkin Elmer, Waltham, MA, USA), setting 64 scans and a resolution of 4 cm^− 1^. ATR-FTIR spectra were collected over the range 4000–700 cm^− 1^. Before the analysis, samples were dried under vacuum at room temperature for at least 24 h. ATR-FTIR analysis (Fig. 7A) confirmed that the artificial ligament was made of PET fibers. All the spectra collected in the central and external regions showed the typical PET patterns, with main adsorption bands centered at 1095 cm^− 1^ (associated with C-O-C symmetric stretching vibration), 1241 cm^− 1^ (associated with C-O asymmetric stretching vibration), and 1720–1725 cm^− 1^ (associated with ester carbonyl stretching [18]. Notably, the broadening of the carbonyl peak and the rise of an absorption band typical of hydroxyl stretching in the 3000–3500 cm^− 1^ region (gray arrows in Fig. 7A, spectrum central 2) indicate a possible depolymerization of PET with the formation of carboxyl end-groups [19]. With regard to the external part of the sample, some regions were highly affected by the presence of biological material. Indeed, the FTIR spectrum also showed the presence of a very intense ester carbonyl peak at 1743 cm^− 1^ (see spectrum external 2 in Fig. 7A), typical of a lipid-rich phase. FTIR analysis of the biological material (bottom spectrum in Fig. 7A) confirmed the lipidic nature of the tissues covering the surface of the graft, with main absorption bands centered at 2925 and 2854 cm^− 1^ (asymmetric and symmetric stretching of C–H of aliphatic methylene group, respectively), 1746 cm^− 1^ (stretching of the ester carbonyl group of the triglycerides, C=O), 1463 and 1458 cm^− 1^ (C-H bending CH_2_ and CH_3_ aliphatic groups), 1162 and 1096 cm^− 1^ (C-O stretching of the ester groups) [20]. Moreover, in this spectrum, a more detailed observation of the complex ester carbonyl stretching band showed a shoulder at about 1735 cm^− 1^ (blue arrow in the bottom right inset of Fig. 7A), indicative of aldehydes [21], possibly derived by peroxidation as a consequence of oxidative stress-induced inflammatory reactions [22].

### Thermal analysis

Differential scanning calorimetric analysis (DSC) was performed on the samples by using a TA-Q2000 differential scanning calorimeter equipped with an RCS-90 cooling unit (TA Instruments). The instrument was calibrated in terms of temperature and energy with pure indium. About 5 mg of the samples were sealed into a Tzero aluminum pan. The measurements were carried out heating samples from 0 to 300 °C at 10 °C/min (I run), then cooling from 300 to 0 °C at 10 °C/m and heating again from 0 to 300 °C at 10 °C/min (II run). High-purity nitrogen gas was fluxed at 20 mL/min during measurements. Melting temperature (T_f_) and related ΔH value (Δ_Hf_) were calculated form the I and II heating runs, while crystallization temperature (T_c_) and the related ΔH value (Δ_Hc_) were calculated from the cooling run. Thermal analysis by DSC was conducted to identify the material constituting the graft and the changes in main thermal parameters associated with tissue infiltration. DSC traces of the central, clean parts of the ligament, and the external, infiltrated part of the ligament are reported in Fig. 7A-D. Calculated thermal parameters are summarized in Supplementary Table 2. The high melting temperature, close to 255 °C almost irrespectively of the region from which the specimens were removed for DCS analysis, confirmed that the artificial ligament was constituted by PET. As shown in Fig. 7B, relevant differences were observed in the glass transition region of the specimens. In more detail, an endothermic wide peak appeared in this region for the external part of the graft, highlighted by the red arrow in Fig. 7B. This phenomenon indicates a physical aging of the amorphous phase, possibly associated with ingrowth and infiltration of the fibrous tissue within the yarn structure of the graft [15]. Moreover, in the high temperature region, peak melting temperatures (T_f_ I run) were similar for both the central and external regions. Instead, a higher enthalpy of fusion (Δ_Hf_ I run) was recorded for the sample taken from the central region of the graft (about 60 J/g with respect to 52 J/g for the external specimen). This suggests a possible increase of crystallinity within the central graft region, eventually associated with a decrease of PET molecular wieght during ageing, as already indicated by ATR-FTIR analysis. This was confirmed by the evaluation of the DSC cooling run, in which, despite the onset crystallization (T_c,onset_) and the peak crystallization temperatures (T_c_) were practically unchanged, crystallization of the central region of the graft was significantly broader, as a possible consequence of partial macromolecular chain fragmentation.

Finally, in the II heating run, melting temperatures (T_f_, II run) and enthalpy of fusion (Δ_Hf_ II run) values of the samples taken from the different regions of the graft were comparable. Moreover, for the external part of the graft, highly infiltrated by the biological tissue, the melting process of the polymer started to be affected by the presence of the degraded biological phases, as shown by the slope of the DSC trace after the melting process (see red arrow in Fig. 7D), indicative of thermal degradation phenomena occurring on PET.

### Revision ACLR surgery

**Fig. 8:**
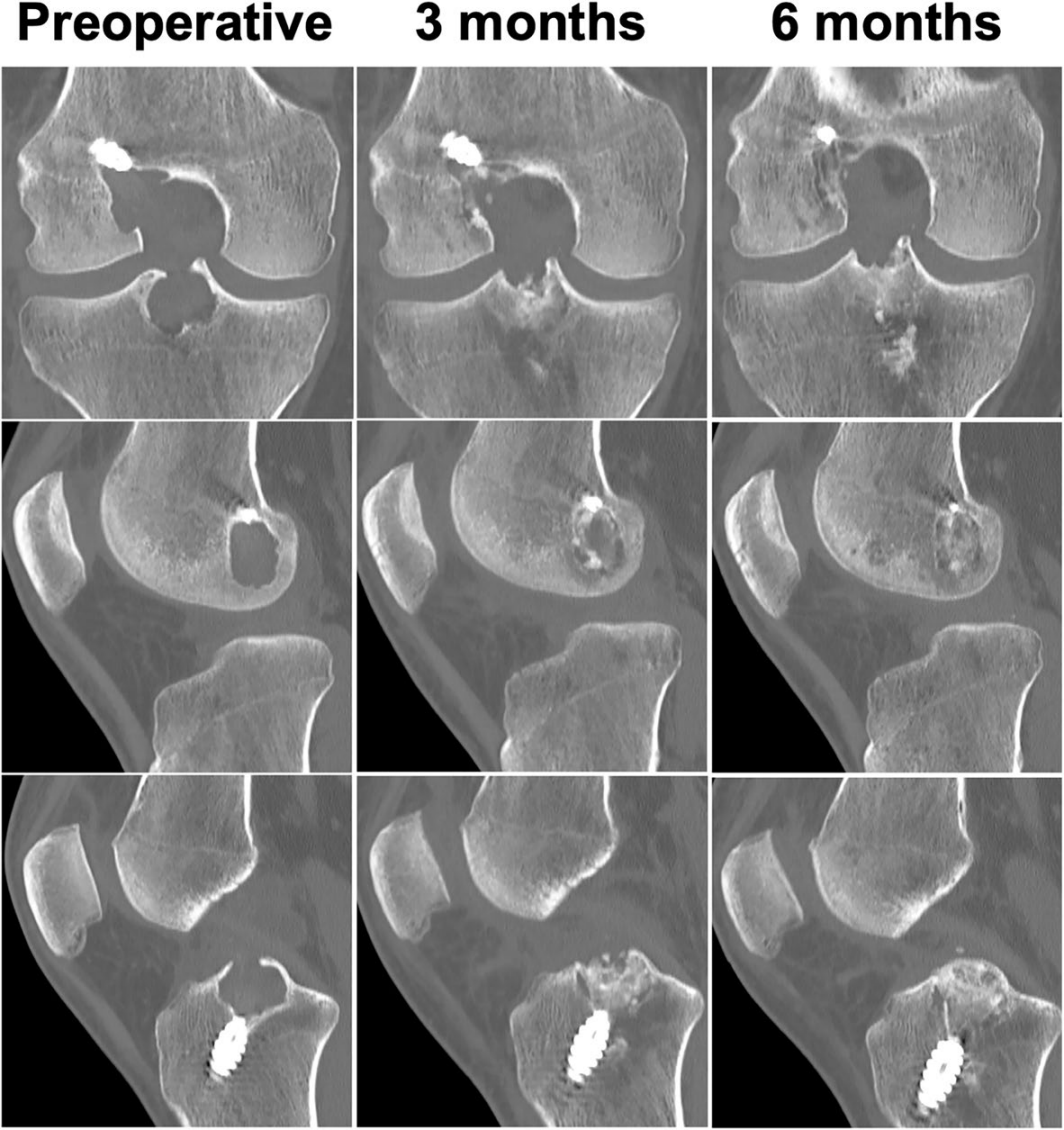
Comparison of preoperative CT scans (left column) with scans acquired at 3 (middle column) and 6 months (right column) after index surgery showing progressive integration of the autologous bone graft in both femoral and tibial tunnels without signs of resorption

The patient was closely observed every month from surgery to month 8, until the knee was pain free, demonstrated a full ROM, and showed no signs of swelling. Furthermore, CT scans of the knee were performed at 3 and 6 months to monitor for bone graft integration (Fig. 8). Eventually, revision ACLR with hamstring tendon autografts was scheduled after 8 months from index surgery. At arthroscopy, there were no signs of effusion and a virtually normal synovium with minimal scar tissue. Following palpation with a probe, healing of the bone graft was confirmed, leaving a cortical layer at both the intercondylar notch and the lateral condyle. The graft was isometrically placed and securely fixed with a button (ULTRABUTTON, Smith&Nephew, London, United Kingdom) and a bioabsorbable tibial screw (BIORCI-HA, Smith&Nephew), without removing pre-existing hardware. The procedure was technically satisfactory. At the time of this publication, the patient has satisfactorily and completely resumed his daily and sport activities (jogging, amateur weightlifting, soccer) at 1 year from the second-stage revision ACLR. A timeline summarizing all the visits and main clinical events is available as Additional File 2.

## Discussion

After a first wave of enthusiasm approximately 30 years ago, the use of artificial ACL grafts has gradually been abandoned to serious issues, including premature failure, bony tunnel osteolysis, wear-debris related synovitis, and early OA [23, 24]. These complications have been confirmed by several in vitro and in vivo studies showing that the release of wear microparticles subsequently activated synoviocytes, macrophages and chondrocytes, eventually stimulating the inflammatory response and the release of cytokines and catabolic enzymes, resulting in synovitis and cartilage degeneration [25]. Nonetheless, the potential advantages associated with the use of synthetic ACL grafts, including lack of donor site morbidity, technically easier and safer surgical technique, and faster patient recovery, have renewed the interest towards a new generation of artificial ligaments, including the LARS [4]. This synthetic graft has a specific design to reproduce the native ACL ultrastructure. Indeed, it consists of two intraosseous segments with longitudinal fibers bounded together by a transverse knitted structure and an intraarticular segment with a side-specific 90°-twisted parallel fiber orientation. Furthermore, the porosity of the material may encourage tissue ingrowth [26] while protecting against friction at the opening of bony tunnels [27]. In a systematic review from Batty et al. [4], the LARS showed lower rates of failure, revision, sterile effusion and synovitis compared to older-generation devices.

Early reports of ACLR with the LARS showed encouraging results, starting from the first study by Dericks et al. in 1995, who reported a success rate of 86% and no case of synovitis among 220 treated patients at a mean follow-up of 2.5 years [28]. Similar outcomes were reported by subsequent short to medium-term studies, with a similar rate of good/excellent results considering knee function, pain, and post-operative complications [29–32]. However, more recent studies at longer follow-ups have demonstrated an alarmingly high rate of major complications. For instance, after a mean follow-up of 9.5 years, Iliadis et al. [10] showed that 31% of patients treated with LARS showed graft mechanical failure. Interestingly, the Kaplan-Meier survivorship curve showed a steep fall of graft failures at approximately 3.5 years. This is in accordance with the study of Tulloch et al. [9], who reported that LARS rupture in his cohort occurred after a median of 3.9 years. Similarly, Tiefenboeck and colleagues [11] reported that 44.4% of patients were subjectively not satisfied, 39% showed signs of OA progression and 56% reported post-operative complications (including superficial and deep infections, graft rupture and recurrent effusions) after a minimum follow-up of 10 years. Smolle et al. [33] have recently published a prospective study with the longest follow-up (median 16.5 years) reported in literature on ACLR with LARS. They reported a cumulative complication rate of 66% (including graft failure and synovitis) and a reoperation rate of 51%.

In this study, we presented a case of a florid foreign body response and disabling synovitis with mechanical failure following ACLR with the LARS in a young active patient. Differently from what has been previously reported, a fibrotic pseudo-myxoid mass restricting the knee ROM developed around the artificial ligament as a result of chronic inflammation, synovial hyperplasia, and foreign body reaction. Similar to previous studies [12, 13, 34], histopathological analysis showed chronic synovial inflammation with the presence of giant multinucleated cells containing PET fibers, thus demonstrating deterioration of the synthetic graft which may have theoretically initiated the inflammatory-foreign body reaction vicious circle. This response has frequently been associated with accelerated OA onset in the previous generation of artificial ligaments and has also been reported in two case reports [24, 35] with LARS, where massive chondrolysis led to premature TKR (one case involving a 23-year-old).

Significant bony tunnel widening due to osteolysis was a prominent finding in this case, with both femoral and tibial tunnels showing a Peyrache grade 3 (> 6 mm) enlargement (Fig. 1F-G) [36]. Similar to Huang et al. [14], the maximum enlargement was noted in proximity of the articular surface, which may be a result of the mechanical instability of the graft as a cause (or a consequence) of the PET-deteriorating inflammatory response [37]. This was also confirmed by the physicochemical analysis performed on the explanted LARS specimen. In fact, both PET depolymerization and formation of lipid peroxidation-derive aldehydes have been documented by our ATR-FTIR analysis. More specifically, the latter have been frequently involved in OA catabolic and inflammatory responses, due to their capacity to trigger chondrocyte apoptosis, induce the release of several metalloproteinases, proinflammatory cytokines, promote the formation of highly immunogenic protein adducts and modify the morpho-functional features of structural matrix proteins [38]. Moreover, PET fragmentation was further confirmed by the increase of crystallinity as suggested by calorimetric analysis, which is indicative of physical aging of the graft. Altogether, fibrous tissue ingrowth, PET depolymerization, increased crystallinity and formation of low-molecular weight byproducts are factors that have been associated with poor biomechanical performances of the graft [15].

With this 2-stage revision approach, joint tissue healing was completely achieved before proceeding with final ACLR using a hamstring graft. At the second surgery, no effusion or cartilage wear were noted, the synovium had healed, and bone grafts had successfully integrated into the previously filled defects, allowing new tunnels to be drilled and secure fixation achieved.

Although a recent metanalysis concluded that ACLR with LARS achieved better postoperative outcomes in terms of restoring knee joint function and stability and was associated with less postoperative complications compared to autografts [39], the use of autografts for primary ACLR remains the ‘gold standard’ in young athletic patients due to higher rates of failure, increased costs, and risk of re-rupture associated with allografts and artificial grafts [40]. Despite the apparent majority of encouraging results, recent evidence suggests that the LARS synthetic ligament may be an alternative graft for ACLR only in selected cases, in one or more of the following conditions: patients > 40 years old; when early recovery is imperative; in the presence of multiligamentous injuries; in revision surgeries in which the availability of autologous tissue for reconstruction is limited [39].

In this case, we encountered a severe foreign body reaction and synovial hyperplasia with osteolysis of both femoral and tibial tunnels following ACLR with LARS. Histological assay, along with advanced morphological and physicochemical analysis were performed to clarify the causes underlying graft failure. These experiments showed signs of PET depolymerization and fibrous tissue infiltration eventually leading to mechanical failure of the graft. This case is a cautionary alert to the use of synthetic ligaments in young and active patients over autografts, whenever available. Indeed, autologous graft have demonstrated optimal biocompatibility and notable biomechanical performance over the last decades, despite donor site morbidity. As history may repeat itself, further use of the LARS device should be cautiously devised.

## Supplementary Information

**Additional file 1.** Summary video Summary video illustrating the main steps of first-stage arthroscopic procedure.

**Additional file 2.** Timeline Timeline summarizing all the visits and main clinical events as per CARE guidelines.

**Additional file 3.** Supplementary Table 1R4

**Additional file 4.** Supplementary Table 2R4

## Data Availability

The datasets used and/or analysed during the current study available from the corresponding author on reasonable request.

## Abbreviations

ACL: Anterior cruciate ligament
ACLR: Anterior cruciate ligament reconstruction
ASIS: Anterior superior iliac spine
ATR: Attenuated Total Reflectance
CT: Computed tomography
DSC: Differential scanning calorimetric analysis
FTIR: Fourier-Transform Infrared
HE: Hematoxylin-eosin
LARS: Ligament Augmentation and Reconstruction System
MRI: Magnetic resonance imaging
OA: Osteoarthritis
PET: Polyethylene terephthalate
ROM: Range of motion
d: Scanning electron microscope
TKR: Total knee replacement
