# Supplementary material for: Massive foreign body reaction and osteolysis following primary anterior cruciate ligament reconstruction with the ligament augmentation and reconstruction system (LARS): a case report with histopathological and physicochemical analysis
Source: BMC Musculoskelet Disord. 2022 Dec 30;23:1140. doi: 10.1186/s12891-022-05984-5 (PMC9801556; doi:10.1186/s12891-022-05984-5)
Supplement: Supplementary file 2 — Additional file 2. Timeline Timeline summarizing all the visits and main clinical events as per CARE guidelines. [file 12891_2022_5984_MOESM2_ESM.pdf]

Timeline

2014-06-29	Index surgery (ACL reconstruction with LARS)
2020-01-15	Right knee X-Ray and MRI
2020-02-03	Sixth post-operative visit: clinical assessment. Final follow-up visit
2020-05-25	First visit at our institution
2020-05-29	Right knee CT is performed to evaluate tunnel osteolysis
2020-06-30	First-stage arthroscopy: debridement, bone grafting and filling of bony defects.
2020-07-13	First post-operative visit: sutures removal and clinical assessment
2020-07-27	Second post-operative visit: clinical assessment
2020-09-07	Third post-operative visit: clinical assessment
2020-09-18	First post-operative right knee CT is performed to evaluate for bone healing
2020-10-18	Fourth post-operative visit: clinical assessment and CT evaluation
2020-11-23	Fourth post-operative visit: clinical assessment
2020-12-22	Fifth post-operative visit: clinical assessment
2021-01-16	Second post-operative right knee CT confirms bone healing
2021-01-20	Sixth post-operative visit: clinical assessment and CT evaluation. Second-stage surgery is planned
2021-02-18	Second-stage arthroscopy: ACL reconstruction with autologous hamstrings
2021-03-04	First post-operative visit: sutures removal and clinical assessment
2021-03-18	Second post-operative visit: clinical assessment
2021-05-15	Third post-operative visit: clinical assessment
2021-08-10	Fourth post-operative visit: clinical assessment. Physical activity is partially resumed
2021-12-12	Fifth post-operative visit: clinical assessment. Return to sport
