# Supplementary material for: Massive foreign body reaction and osteolysis following primary anterior cruciate ligament reconstruction with the ligament augmentation and reconstruction system (LARS): a case report with histopathological and physicochemical analysis
Source: BMC Musculoskelet Disord. 2022 Dec 30;23:1140. doi: 10.1186/s12891-022-05984-5 (PMC9801556; doi:10.1186/s12891-022-05984-5)
Supplement: Supplementary file 3 — Additional file 3. Supplementary Table 1R4 [file 12891_2022_5984_MOESM3_ESM.docx]

Supplementary Table 1. Laboratory values of the patient on admission before

| **Laboratory parameter (unit of measure)** | **Value** | **Normal range** |
| --- | --- | --- |
| Hb (g/dL) | 16.70 | 13.50 – 17.50 |
| RBC (x10^6^/μl) | 5.37 | 4.30 – 5.50 |
| WBC (x10^3^/μl) | 8.25 | 4.00 – 10.00 |
| HCT (%) | 44.50 | 40.00 – 50.00 |
| MCV (fl) | 82.90 | 83.00 – 101.00 |
| RDW (%) | 12.90 | 11.00 – 16.00 |
| PLT (x10^3^/μl) | 215.00 | 150.00 – 400.00 |
| AST (U/L) | 32.00 | 5.00 – 34.00 |
| ALT (U/L) | 51.00 | 0.00 – 55.00 |
| GGT (U/L) | 14.00 | 11.00 – 59.00 |
| Plasma sodium (mmol/L) | 140.00 | 136.00 – 145.00 |
| Plasma potassium (mmol/L) | 3.30 | 3.50 – 5.10 |
| Creatinine (mg/dL) | 0.84 | 0.73 – 1.18 |
| Plasma glucose (mg/dL) | 105.00 | 74.00 – 106.00 |
| Blood urea (mg/dL) | 24.40 | 19.00 – 43.00 |
| APTT (seconds) | 32.70 | 23.00 – 32.00 |
| INR | 1.11 | 0.80 – 1.20 |
| CPR (mg/dL) | 0.05 | < 0.50 |

ALT = alanine transaminase; APTT = activated partial thromboplastin time; AST = aspartate transaminase; CRP = C reactive protein; GGT = gamma-glutamyltransferase; Hb = hemoglobin; INR = international normalized ratio; MCV = mean corpuscular volume; PLT = platelets; RBC = red blood cells; RDW = red blood cells distribution width; WBC = white blood cells.
