# Supplementary material for: Massive foreign body reaction and osteolysis following primary anterior cruciate ligament reconstruction with the ligament augmentation and reconstruction system (LARS): a case report with histopathological and physicochemical analysis
Source: BMC Musculoskelet Disord. 2022 Dec 30;23:1140. doi: 10.1186/s12891-022-05984-5 (PMC9801556; doi:10.1186/s12891-022-05984-5)
Supplement: Supplementary file 4 — Additional file 4. Supplementary Table 2R4 [file 12891_2022_5984_MOESM4_ESM.docx]

**Supplementary Table 2**. Thermal parameters calculated from DSC analysis of the central and external part of the explanted LARS graft.

|  | T_f_ I run (°C) | ΔH_f_ I run (J/g) | T_c,onset_ (°C) | T_c_  (°C) | ΔH_c_  (J/g) | T_f_ II run (°C) | ΔH_f_ II run (J/g) |
| --- | --- | --- | --- | --- | --- | --- | --- |
| central | 255.5 | 60.3 | 219.4 | 213.6 | 54.2 | 254.1 | 45.0 |
| external | 253.6 | 51.9 | 219.9 | 213.7 | 51.3 | 249.1 | 43.9 |
